# Postharvest Quality of Cherry Tomatoes Coated with Mucilage from Dragon Fruit and Irradiated with UV-C

**DOI:** 10.3390/polym13172919

**Published:** 2021-08-30

**Authors:** Zuliana Razali, Chandran Somasundram, Siti Zalifah Nurulain, Wijenthiran Kunasekaran, Matthew Raj Alias

**Affiliations:** 1 Institute of Biological Sciences, Faculty of Science, University of Malaya, Kuala Lumpur 50603, Malaysia; chandran@um.edu.my (C.S.); ainzalifah@gmail.com (S.Z.N.); matthewraj111@gmail.com (M.R.A.); 2 The Center for Research in Biotechnology for Agriculture (CEBAR), University of Malaya, Kuala Lumpur 50603, Malaysia; 3 Cytonex Sdn Bhd, Kuala Lumpur, Federal Territory of Kuala Lumpur, Kuala Lumpur 51200, Malaysia; wijen@cytonexlab.com

**Keywords:** cold storage, edible coating, mucilage, postharvest shelf life, cherry tomato, microbial analysis

## Abstract

Cherry tomatoes are climacteric fruits that have a limited shelf life. Over the years, many methods have been applied to preserve the fruit quality and safety of these fruits. In this study, a novel method of combining mucilage from dragon fruits and UV-C irradiation was carried out. Cherry tomatoes were subjected to UV-C irradiation and edible coating, both as a stand-alone and hurdle treatment. The edible coating was prepared from the mucilage of white dragon fruits. Quality parameters including color, weight loss, total soluble solids, titratable acidity, ascorbic acid, antioxidant analysis (total phenolic content and flavonoid content), and microbial analysis were measured throughout 21 days of storage at 4 °C. Results showed that the hurdle treatment extended shelf life by 21 days, reduced weight loss (0.87 ± 0.05%) and color changes (11.61 ± 0.95 ΔE), and inhibited microbes better than stand-alone treatments. Furthermore, fruits treated with the combination of UV-C and edible coating also contained higher total polyphenol content (0.132 ± 0.003 mg GAE/100 mL), total flavonoid content (13.179 ± 0.002 mg CE/100 mL), and ascorbic acid (1.07 ± 0.06 mg/100 mL). These results show that the combination of UV-C and edible coating as a hurdle treatment could be an innovative method to preserve shelf life and quality of fruits.

## 1. Introduction

The consumption of fresh fruits has sharply increased in the past century, which triggered a commercial demand for better logistical and warehousing elements to preserve the quality of fresh produces (e.g., flavor, color, nutritional aspects, shelf life, and processing characteristics), at the same time controlling the spread of postharvest diseases of fresh produce during its shelf life [1].

Tomatoes have become a staple commodity to humankind because they are rich in carotenoids, polyphenols, and vitamin C [2]. Moreover, tomatoes are also consumed for their high lycopene content (71.6%), pro-vitamin A carotenoids (14.6%), beta-carotene (17.2%), vitamin E (6.0%), and lastly as a crucial source of vitamin C [3]. One of the most popular tomatoes is cherry tomatoes, which are aromatic and red-colored, with a hard texture and small size [4], and known worldwide for their nutritional value and taste [5].

Although tomatoes have a plethora of nutritional and antioxidant attributes, they are plagued with a short shelf life. Tomatoes are highly perishable due to their climacteric nature [6]. Temperature and humidity control the rate at which tomatoes ripen, eventually rendering the fruit inedible due to softening of the flesh [7].

As a result, many methods have been introduced to prolong the shelf life of tomatoes. The postharvest disease was controlled using synthetic chemicals; however, due to health and environmental concerns posed by fungicide deposits, stringent regulations have been imposed regarding their use [8]. Alternative methods such as controlled atmosphere [9] and ethanol vapor treatment [10] have extended the shelf life of tomatoes.

A more innovative approach towards reducing postharvest losses is using an edible coating [11] and prolonging the shelf life of agricultural products [12]. A study conducted was also able to prolong the shelf life of cherry tomatoes by pullulan coating with ethanol extract of propolis during refrigerated storage [13]. Coatings act as barricades during handling, processing, and storage and do not exclusively delay food deterioration but also enhance the quality of the product. Moreover, they are also safe due to the integration of antimicrobial compounds or the coating’s natural biocide activity [14].

A study conducted by [15] managed to extend the shelf life of tomatoes by coating the tomatoes with alginate reinforced with titanium oxide nanoparticles (*n*TiO_2_) followed by irradiation with UV light. Similar studies by [16] highlighted that UV-B + UV-C with edible coating on sweet cherry fruit significantly reduced weight loss. The combination of UV-B + UV-C on the fruit with edible coating also increased vitamin C and total phenolic compounds and showed higher retention for anthocyanin and total antioxidant compared to untreated fruit.

Conversely, UV-C radiation can be administered at a low cost, and it is less harmful to the environment, which allows it to be used as an alternative compared to fungicides [16,17]. The UV-C mechanism could prevent the onset of decay via an antimicrobial effect. Exposure to UV-C induces the production of phenols which are toxic to pathogens [18]. Moreover, UV-C affects fruit metabolism, predominantly on cell wall metabolism [18,19,20], activation of antioxidant enzymatic systems, production of pathogenesis-related (PR) proteins, and biosynthesis of antioxidant and antifungal compounds such as terpenoids, phenolics, ascorbic acid and folates, and polyamines [16,19,21,22,23]. However, a combination of UV-C and edible coating derived from dragon fruit mucilage has yet to be done, which could provide a low-cost method in improving the postharvest quality of cherry tomatoes. While there are studies on the use of edible coating and UV irradiation, there is a lack of literature on the use of high antioxidant plant-based coating material in combination with UV-C. Hence, this study investigates the effect of dragon fruit mucilage coating and UV-C on cherry tomatoes’ shelf life and physicochemical and nutritional quality.

## 2. Materials and Methods

### 2.1. Extraction of Plant Mucilage

Fresh white dragon fruits (*Hylocereus undatus*) purchased from a local supermarket were washed under running water to remove dirt and debris. Then, the skin was peeled, and the pulp was removed. The hydration method [24] with slight modifications was used to extract the mucilage from the pulp. Firstly, the separated fruit pulp was weighed and then forced through a sieve with a pestle to remove the seeds. The volume of seedless pulp was measured with a measuring cylinder. Ethanol was added with the ratio of 2:3 (extract: alcohol) to precipitate the mucilage for 24 h (hour) at 4 °C and collected by filtration and then oven-dried for 24 h at 40 °C.

### 2.2. Treatment of Cherry Tomatoes

**Figure 1:**
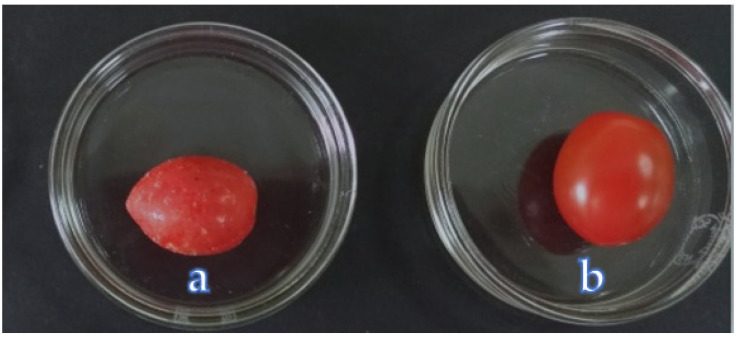
Cherry tomato coated with mucilage (**a**); uncoated cherry tomato (**b**).

**Figure 2:**
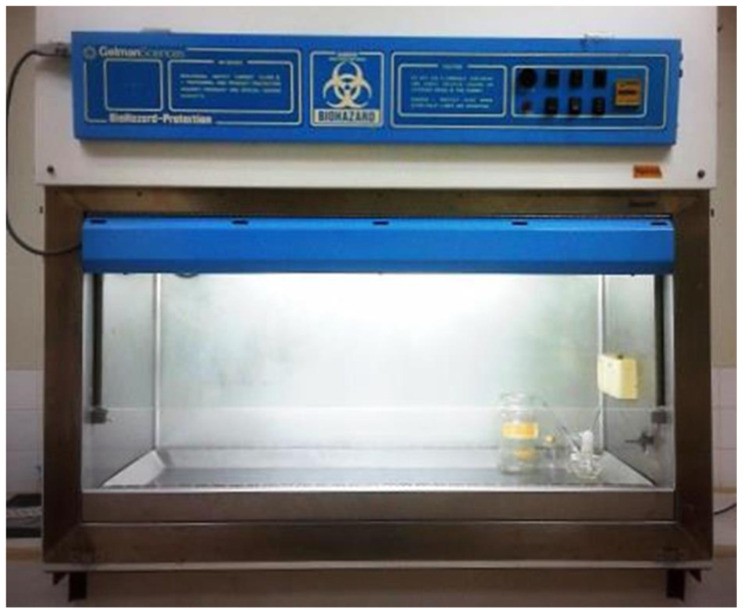
UV-C lamp in a laminar flow cabinet.

Cherry tomatoes (*Solanum lycopersicum var. cerasiforme*) were washed using distilled water and dried at room temperature. From a pool of cherry tomatoes, 25% of them were used as control (Figure 1b) (group A); 25% were dipped in a solution consisting of pure mucilage diluted threefold with distilled water for 30 s (Figure 1a) (group B); 25% of cherry tomatoes in group C were irradiated by fluorescent germicidal lamps (Figure 2), 15 cm away from the surfaces of the lamp for 8 min (minutes). Another 25% of cherry tomatoes (group D) were also irradiated by fluorescent germicidal lamps under the same condition as above and subsequently dipped in a solution comprising pure mucilage diluted with distilled water (1:3) for 30 s. The coated fruits were air-dried (28 °C) for 30 min. All the fruits were stored at 4 °C at 95% relative humidity. The fruits were observed and evaluated on days 0, 3, 7, 14, and 21 based on their color, weight loss, total phenolic contents, total flavonoid content, ascorbic acid (DCPIP) soluble solids, titratable acidity, and microbial analysis.

### 2.3. Color Measurement

The color was determined using Chroma Meter (Minolta Chroma Meter CR-200, Minolta, Osaka, Japan) with CIE LAB color scale (L*, a* and b* values). The instrument was calibrated using a white reference tile. The color parameters that consist of L* (lightness/darkness), a* (redness/greenness), and b* (yellowness/blueness) were evaluated. Color differences (∆E) were calculated [25] to compare with control samples using Equation (1).

∆E = [(∆L*)^2^ + (∆a*)^2^ + (∆b*)^2^] ^½^ (1)

### 2.4. Weight Loss

Tomato samples were weighed using a Mettler Toledo (SB16001) weighing balance on days 3, 7, 14, and 21. The difference between initial and final fruit weight was considered as total weight loss during each storage interval and calculated as percentages on a fresh-weight basis by the standard AOAC (2010) method [26].

### 2.5. Antioxidant Analysis

#### 2.5.1. Preparation of Extract

Samples for antioxidant analysis were extracted according to [27] with slight modifications. To purify the sample, equal parts of tomato juice were added to 80% methanol (5 mL:5 mL). The mixture was placed in a shaking incubator (Shellab Orbital Shaking Incubator S14, Cornelius, OR, USA) at 250 rpm for 30 min at room temperature followed by centrifugation (Beckman J2-MI Centrifuge, Ramsey, MN, USA) at 5 °C and 6500 rpm for 15 min. The precipitate was discarded, and the remaining supernatant was filtered using a steel sieve with an approximate diameter of 2 mm to obtain juice and subsequently stored in sterile glass bottles. The supernatant was used for the analysis of antioxidant activity.

#### 2.5.2. Total Polyphenol Content

The total polyphenol content was determined using Folin–Ciocalteu assay [26] modified to a microscale [28]. Juice extract and gallic acid standard solution (10 µL) were added to 790 µL SDW and 50 µL Folin–Ciocalteu reagent (Sigma-Aldrich, Petaling Jaya, Malaysia) in a 1.5 mL microcentrifuge tube and mixed. After 1 min, 150 µL of 20% sodium carbonate solution was added, and the solution was mixed by inverting the tubes. The mixture was allowed to stand at room temperature (25 ± 1 °C) for 120 min (in the dark). Absorbance was measured at 750 nm (UV-200-RS Spectrophotometer, MRC, Holon, Israel) against a prepared blank (replace juice extract with SDW). A standard curve of gallic acid (y = 0.9078x, r^2^ = 0.9977 check with paper) was prepared, and results were reported as milligrams of gallic acid equivalent (GAE) per 100 mL cherry tomato juice extract.

#### 2.5.3. Total Flavonoid Content

The flavonoid content of cherry tomato juice samples was determined using a colorimetric method described by [29]. A standard curve of (+)-catechin (y = 0.0028x, r^2^ = 0.9977) was prepared and results were reported as milligrams of catechin equivalent (CE) per 100 mL juice extract.

#### 2.5.4. Ascorbic Acid Content

The ascorbic acid content in samples was determined based on the 2,6-dichlorophenol-indophenol (DCPIP) visual titration method [30]. Cherry tomato juice was diluted with 3% metaphosphoric acid and filtered. Then, the filtrate was titrated with standardized dye solution (2,6-dichloroindophenol-indophenol and sodium bicarbonate) to a pink endpoint. The results obtained were expressed as milligrams of ascorbic acid per 100 mL sample using Equation (2).

(2) $$Ascorbic\;acid\;content\;=\frac{titre\times dye\;factor\times volume\;made\;up\times 100}{aliquot\;of\;extract\;taken\;for\;estimation\times volume\;of\;sample\;taken\;for\;estimation}.$$

### 2.6. Total Soluble Solid

Total soluble solids (TSS) were determined using a digital refractometer (Atago PR-1 digital refractometer, Tokyo, Japan) at 25 ± 1 °C, and results were expressed in a standard °Brix unit.

### 2.7. Titratable Acidity

To determine titratable acidity, diluted cherry tomato juice was titrated with standardized 0.1 *N* sodium hydroxide to a definite faint pink endpoint using phenolphthalein as an indicator. The volume of sodium hydroxide used for titration was converted to grams of citric acid per 100 mL of juice according to the method in [31]. The titratable acidity (%*TA*) was calculated using Equation (3):

(3) $$\%TA=\frac{V_{1}\times 0.1NaOH\times Eq.\;wt.\times 100}{V_{2}\times 1000}$$

where *V*_1_ is the volume of titrant (mL), *Eq. wt.* is the equivalent weight of anhydrous citric acid (64 mg/mEq), and *V*_2_ is the volume of sample (mL).

### 2.8. Microbial Inactivation Analysis

The 3M Petrifilm plate methods are recognized as AOAC International Official Methods of Analysis (3M Food Safety, 2010). Microbial count of juice samples was determined using Petrifilm plates (3M Center, St. Paul, MN, USA) for aerobic bacteria, coliform, yeast, and mold and were calculated as colony-forming units [32]. The results were expressed as log (CFU/mL).

### 2.9. Statistical Analysis

Data obtained were subjected to statistical analysis using SPSS 22.0 software (SPSS Inc., IBM New York, NY, USA). In this study, data were represented as mean values ± standard error (SE) (*n* = 12). The significant differences between mean values of juice samples were determined by analysis of variance (one-way ANOVA) using Tukey’s honestly significant difference (HSD) test at a significance level of *p* < 0.05.

## 3. Results and Discussion

### 3.1. Color Analysis

**Figure 3:**
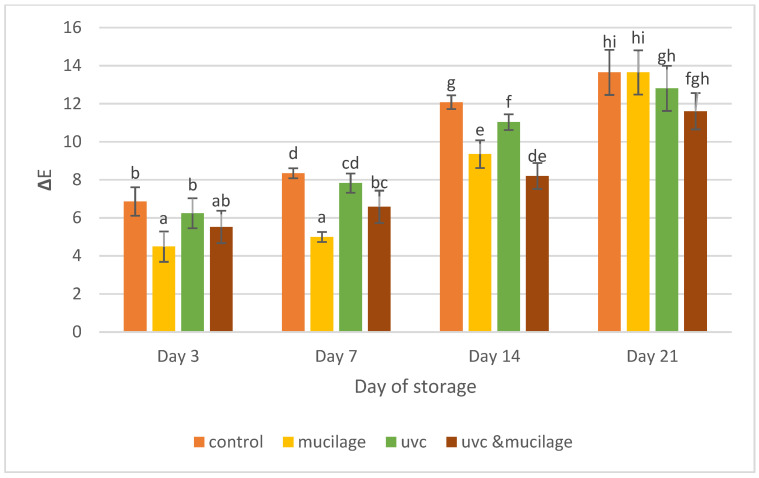
Effect of mucilage coating and UV-C on color. Values followed by different letters within the same column are significantly different (*p* < 0.05) (*n* = 12).

The color parameters for different samples were evaluated as ΔE and presented in Figure 3. Results showed that the values significantly increase from Day 3 to Day 21 for all samples. The control sample showed the highest result of color change on Day 21 (13.65 ± 1.18 ΔE) from Day 0, while the hurdle treatment showed the least color change on Day 21 (11.61 ± 0.95 ΔE) from day 0. ΔE is calculated based on the L* (lightness; 0 = black, 100 = white), a* (+a = redness, −a = greenness), and b* (+b = yellowness, −b = blueness).

Color parameters can be grouped as not noticeable (0 < ΔE < 0.5), slightly noticeable (0.5 < ΔE < 1.5), noticeable (1.5 < ΔE < 3.0), well visible (3.0 < ΔE < 6.0), and greatly visible (6.0 < ΔE < 12) [33]. There is a significant difference in ΔE of all samples ranging from 4.49 ΔE to 13.65 ΔE. During tomato ripening, chlorophyll is degraded from green to colorless compounds, and at the same time, carotenoids are synthesized from a colorless precursor (phytoene) to carotene (pale yellow), lycopene (red), β-carotene (orange), and hydroxylated carotenoids (yellow) [34].

The increasing data showed the result of the ripening process where fruit color will be redder and darker. The hurdle treatment sample maintained the typical red, bright color for 21 days of storage under 4 °C. All treatment samples were better than the control sample during storage. As mentioned earlier, hurdle treatment samples have the slightest color change compared to other samples. This shows that the hurdle treatment keeps the appearance of fruits in the best of conditions.

### 3.2. Weight Loss

**Figure 4:**
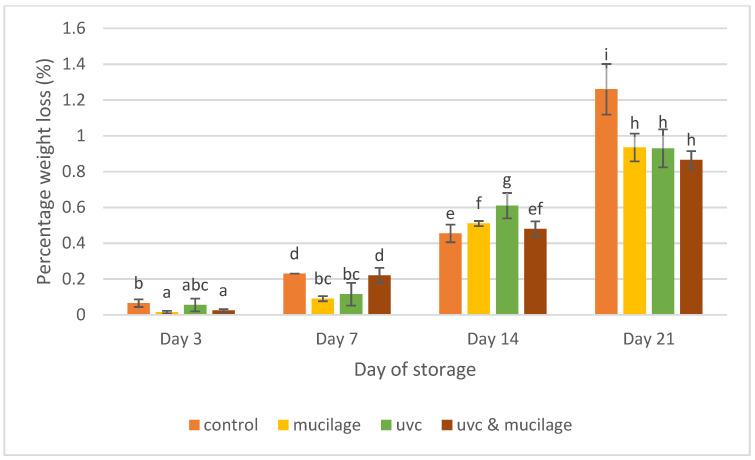
Effect of mucilage coating and UV-C on percentage weight loss. Values followed by different letters within the same column are significantly different (*p* < 0.05) (*n* = 12).

Figure 4 shows that the percentage of weight loss for all samples was not significant during the first seven days but increased significantly after storage at 4 °C. After 21 days, the control sample lost more weight (1.26 ± 0.14%), while the hurdle treatment sample has the least weight loss (0.87 ± 0.05%). The weight loss showed an increasing trend during a prolonged storage period in both treated and untreated samples. Mucilage as an edible coating can delay the migration of fruits because the coating can reduce respiration and transpiration, resulting in the lowest weight loss percentage. The coating acts as a physical barrier that helps to reduce moisture loss, solute movement, and gaseous exchange (O_2_ and CO_2_) due to the formation of a film/coating on top of the skin [35]. Similar results were reported by [36], where peaches coated with rhubarb-SA were found to have lower levels of weight loss. Correspondingly, another study found that beeswax coatings decreased the respiration rate of the fruits, thus reducing the weight loss and increasing the shelf life of sweet orange [37]. Samples exposed to UV-C irradiation for 10 to 15 min resulted in smaller weight loss than the control [38]. Thus, combining two treatments as a hurdle treatment was the most effective in reducing the percentage weight loss of cherry tomatoes during storage.

### 3.3. Titratable Acidity

**Figure 5:**
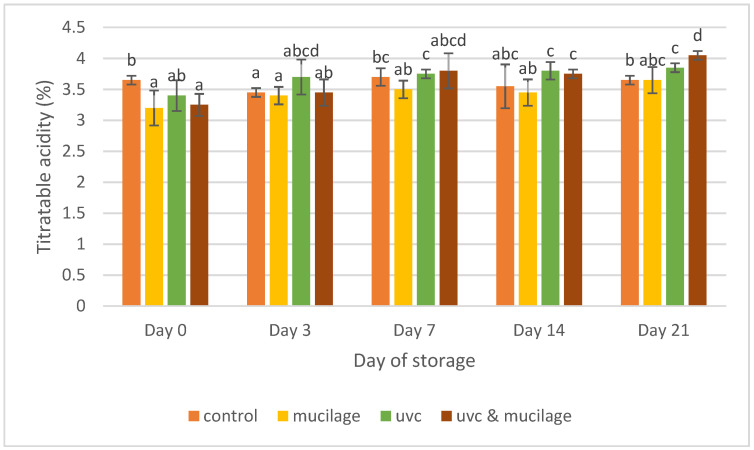
Effect of mucilage coating and UV-C on titratable acidity. Values followed by different letters within the same column are significantly different (*p* < 0.05) (*n* = 12).

Figure 5 shows the results of titratable acidity for different types of treatments on cherry tomatoes. While the graph shows an increasing trend, the titratable acidity in all the treated samples was higher than the control at the end of the 21-day storage period. This can be explained by the fact that the coating may have slowed the respiration rate of the fruits; thus, the rate of utilization of respiratory substrate was very minimal [39]. Similar results were reported [40]; there were slightly higher titratable acidity values after UV-C light on coconut water. Thus, this combination treatment resulted in the highest results at the end of the storage period.

### 3.4. Total Soluble Solids

**Figure 6:**
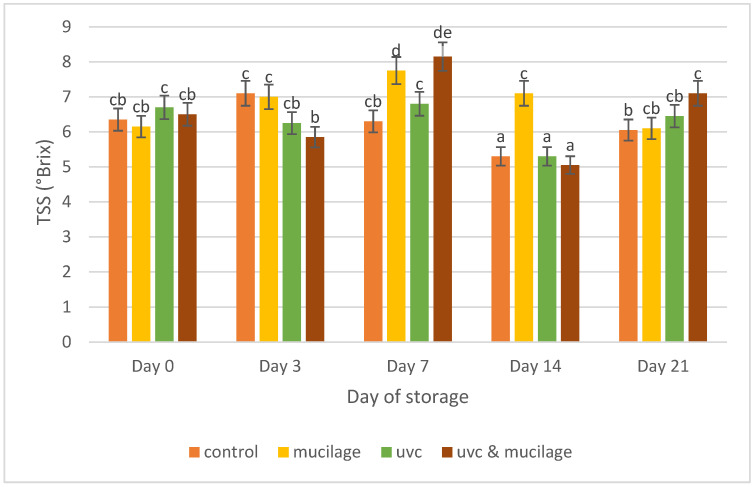
Effect of mucilage coating and UV-C on total soluble solids. Values followed by different letters within the same column are significantly different (*p* < 0.05) (*n* = 12).

Total soluble solid content is an important quality indicator to measure the sweetness of cherry tomatoes. Figure 6 shows an increasing trend for TSS (°Brix) from day 0 to 7, and it decreased after that. This could result from increasing sugars during the storage period through the degradation of polysaccharides [41]. The ripening of cherry tomatoes is caused by the degradation of organic acids and the accumulation of sugars during storage [42]. However, in our study, the UV-C irradiated sample did not show much difference throughout 21 days of the cold storage period. This could be because fruits used in this experiment were harvested at the mature red stage, which reduced the effect of UV-C on the total soluble solids. The fruit already had undergone biochemical processes and accumulated all the sugars, causing results that were not comparable [43]. Similar results were reported in [44], which found that UV-C irradiation had a minimal effect of total soluble solid on ripe tomatoes for 21 days of storage at 20 °C.

Furthermore, UV-C treated samples have lower sugar content than the control. Samples from all treatments were higher than the control for day 21 [42], and these results agreed with those obtained in [45]. Hurdle treatment had the highest TSS (°Brix) content at the end of storage and was the most effective way to increase the sugar contents.

### 3.5. Antioxidant Analysis

**Figure 7:**
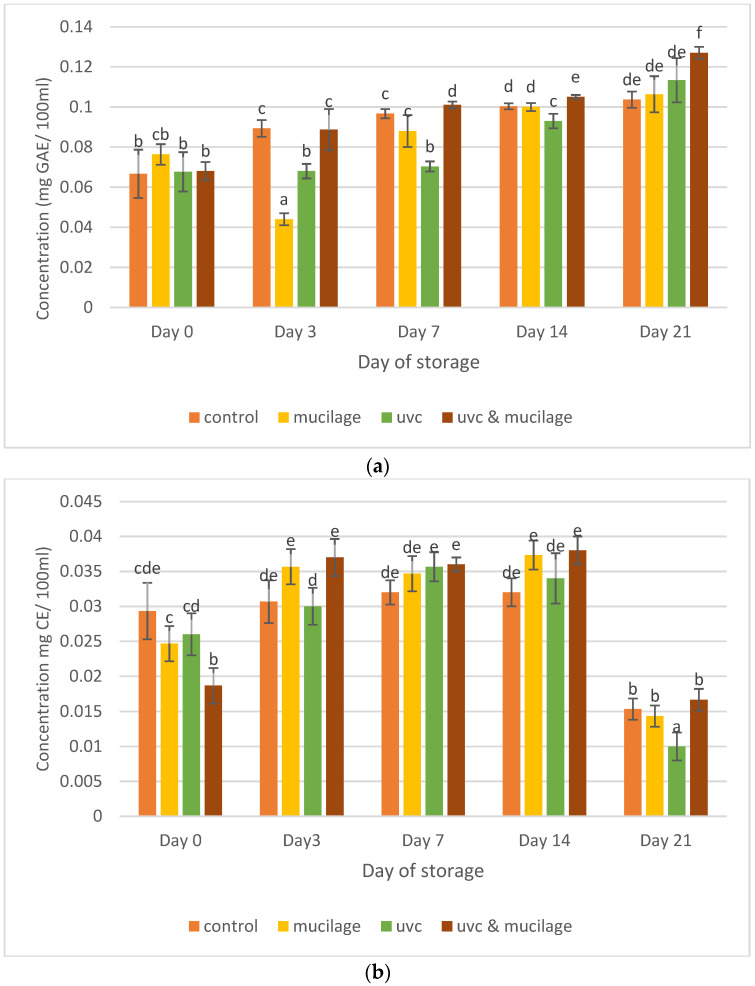
Effect of mucilage coating and UV-C on (**a**) total phenolic content and (**b**) total flavonoid content. Values followed by different letters within the same column are significantly different (*p* < 0.05) (*n* = 12).

Figure 7a shows the effect of mucilage coating and UV-C on total phenolic content for all samples. The results show a general increase from Day 0 to Day 21. The hurdle treatment sample’s highest antioxidant activity was recorded on Day 21 with 0.132 ± 0.003 (mg GAE/100 mL) and the least by the mucilage-treated sample on Day 3 with 0.04 ± 0.003 (mg GAE/100 mL). The effect of mucilage coating and UV-C on total flavonoid content is presented in Figure 7b. The results show an increasing trend from Day 0 to 21, but a significant reduction was observed for all samples on Day 21. The hurdle treatment sample showed the highest antioxidant activity on Day 14 with 13.179 ± 0.002 (mg CE/100 mL). This sample also showed the highest activity on Day 21, even though there was a reduction for all samples.

The application of edible coatings to fresh fruit has been associated with an accumulation of phenolic compounds and ascorbic acid, causing an increase in the fruit’s antioxidant capacity [46]. Previous studies have shown that low O_2_ and high CO_2_ concentrations increased the production of phenolic compounds during storage in fresh-cut melons [47]. An increase in phenylalanine ammonia-lyase (PAL) enzyme is responsible for synthesizing phenolic compounds in grapes [48]. In addition, UV-C can result in the release of bound phenolic contents and the activation of PAL enzymes responsible for synthesizing some phenolic compounds such as flavonoids, chlorogenic acids, coumarins, and phenylpropanoids [49].

Similarly, total flavonoid content decreased during cold storage of various cultivars of hardy kiwifruits during cold storage [50]. Edible coatings can produce abiotic stress on produce, modify its metabolism, and affect the production of secondary metabolites such as phenolics and flavonoid compounds [51]. In this study, hurdle treatment was the most effective way to increase the antioxidant activity for total phenolic and flavonoid content during postharvest storage.

### 3.6. Ascorbic Acid Content

**Figure 8:**
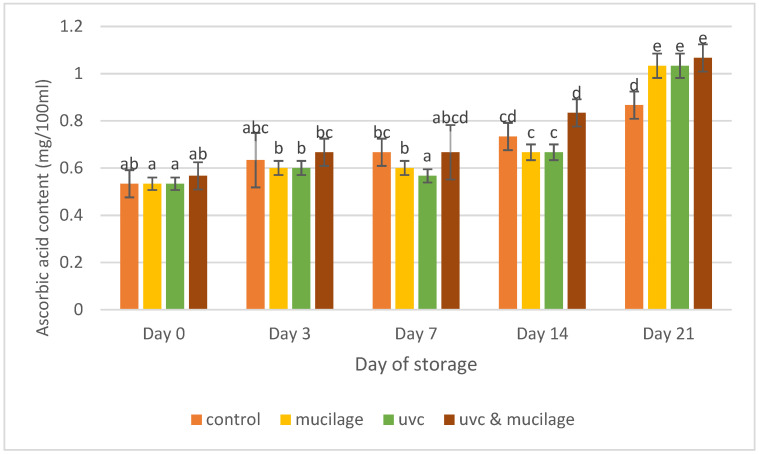
Effect of mucilage coating and UV-C on the ascorbic acid content. Values followed by different letters within the same column are significantly different (*p* < 0.05) (*n* = 12).

The effect of mucilage coating and UV-C on the ascorbic acid content of the samples is shown in (Figure 8). The ascorbic acid content increased throughout the experiment. Hurdle treatment showed the highest ascorbic acid content (1.07 ± 0.06 mg/100 mL) on Day 14. The stability of ascorbic acid in fruits is usually influenced by high titratable acidity [52]. Ascorbic acid content increases with ripening and storage time; however, the content declines once the fruit is fully ripe [53]. The mucilage-coated sample showed a gradual increase in ascorbic acids during the storage period. This suggests that that treatment did not prevent the synthesis of ascorbic acid content during the ripening process. In addition, UV-C treatment can increase the levels of ascorbic acid in fruits; as reported, it can modulate several vital antioxidant enzyme activities in cherry tomatoes [51].

### 3.7. Microbial Analysis

**Figure 9:**
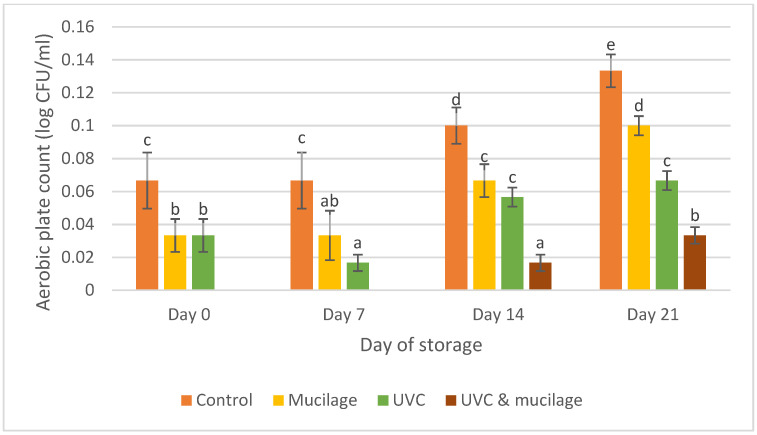
Effect of mucilage coating and UV-C on microbial analysis. Values followed by different letters within the same column are significantly different (*p* < 0.05) (*n* = 12).

Figure 9 shows the effect of mucilage coating and UV-C on microbial analysis for all samples. The results showed a significant increase from Day 0 to 21. The hurdle treatment sample showed no microbial activity on Day 0 and 7 and had the least microbial activity compared to other samples on Day 14 and 21. The control sample showed the highest activity on Day 21 with 0.133 ± 0.010 (log CFU/mL). The result from this study is agreeable to that of [54], which reported that coating could extend shelf life by limiting the growth of bacteria or fungi and decreased the spoilage without affecting the ripening process of fruits. Mucilage will create a layer to prevent bacteria or foreign substances from entering the sample’s tissue. The coating materials extracted from dragon fruits were also antioxidant and antimicrobial, thus reducing the microorganisms’ attack and resulting in longer shelf life [55].

UV-C light will kill or inactivate microorganisms by destroying nucleic acids and disrupting their DNA, leaving them unable to perform their functions. Furthermore, UV-C can also reduce microbial activity by killing bacteria through the germicidal effect. The resistance of UV-C-treated cherry tomatoes to decay is linked to the synthesis and accumulation of phytoalexins, which are antimicrobial secondary metabolites [56]. Moreover, another study also showed that the combination of UV irradiation and edible coating induced a synergetic effect that improved shelf life and food quality [57]. In this study, hurdle treatment effectively delays the ripening process and extends cherry tomatoes’ storage life.

## 4. Conclusions

This study showed that the hurdle treatment was the best in extending shelf life and inhibiting microorganisms due to the potent effect of UV-C irradiation on microorganisms that cause fruit decay together with the antimicrobial and antioxidant effect of the mucilage coating. The hurdle treatment also effectively reduced weight loss due to the mucilage barrier, which limits water transpiration and respiration. Color was slightly altered by the hurdle treatment, therefore maintaining the visual appearance of the fruit even after 21 days in storage. Furthermore, fruits treated with a combination of UV-C and edible coating also contained higher ascorbic acid content and antioxidant capacity. These results demonstrate that the combination of UV-C and edible coating as a hurdle treatment could be an innovative method to preserve shelf life and quality of fruits.

## Data Availability

Not applicable.
